# Perineural invasion and laryngeal squamous cell carcinoma: a systematic review

**DOI:** 10.1016/j.bjorl.2024.101519

**Published:** 2024-11-01

**Authors:** Debora Modelli Vianna Ocampo Quintana, Rogerio Aparecido Dedivitis, Luiz Paulo Kowalski

**Affiliations:** a Hospital Heliópolis, Departamento de Cirurgia de Cabeça e Pescoço, São Paulo, SP, Brazil; b Hospital Das Clínicas da Faculdade de Medicina da Universidade de São Paulo (HC-FMUSP), Departamento de Cirurgia de Cabeça e Pescoço, São Paulo, SP, Brazil

**Keywords:** Laryngeal cancer, Squamous cell carcinoma, Perineural invasion, Prognosis, Systematic review

## Abstract

**Objectives:**

Several studies have evaluated the prognostic significance of Perineural Invasion (IPN) in laryngeal cancer; however, the results are non-conclusive. Therefore, we conducted a meta-analysis aiming to identify the prognostic value of IPN in laryngeal cancer.

**Methods:**

A literature review was performed, searching MedLine via PubMed, Scielo, Lilacs, Cochrane and Websco. RevMan 5.4 was used for the statistical analysis.

**Results:**

A total of 19 laryngeal cancer studies were included. The results indicate that IPN in laryngeal cancer shows 2-year survival with RR 0.71 (0.59‒0.86); disease-free survival RR 0.51 (0.14–1.95); and locoregional recurrence RR 1.71 (1.25–2.35).

**Conclusion:**

IPN is a negative prognostic factor in laryngeal cancer.

## Highlights

• IPN is an independent factor for increased mortality in laryngeal cancer.
• Meta-analysis concludes that IPN is causal factor for reduced survival and increased relapse.
• Meta-analysis indicates need for standardization of definition and the study of IPN in future articles.

## Introduction

In 2021, the estimated deaths for laryngeal cancer were 3,770, with the incidence of new cases estimated at 12,620; from 2015 to 2019, the mortality rate was 0.9/100,000 [1]. The 5-year survival rate varies according to the initial tumor staging and its subsite. Prognosis depends on lesion size, level of local invasion, regional lymphatic dissemination and presence of distant metastases [2]; other predictors are race, gender, vascular embolization, Perineural Invasion (IPN) and histological grade [3]. The evaluation of prognostic factors acts as a guide for the treatment and follow-up of neoplasms [4].

## Literature review

IPN is a form of neoplastic dissemination of neurotropic tumors, usually related to aggressive behavior, recurrence, and increased morbidity and mortality [5]. It has been described in several neoplastic sites, such as pancreas, prostate, colorectal, and head and neck [6], and was first recognized in 1835 by Cruveillheir [7], but defined in the specialty in 1985 by Batsakis [8], in a generic way. It was only in 2009 that Liebig et al. [5] brought a more specific definition, noting IPN as “invasion of one of the three nerve layers or the involvement of at least one third of the circumference of the nerve”.

IPN may be difficult to diagnose ‒ due to the presence of perineural inflammatory cells that make its microscopic analysis difficult ‒ or due to the possibility of niches of neoplastic cells being found erratically inside the nerve (“skip lesions”), making its microscopic diagnosis difficult [9].

A study by Shen et al. [10] identifies IPN in 22% of pathology specimens, with this proportion increasing to 39% in slide reviews and to 51% in reviews associated with immunohistochemical studies. Besides the observational issue, the way the slide is prepared also influences its diagnosis; longitudinal slides tend to identify more intraneural invasion and “skip lesions”; transverse slides tend to identify circulatory contact with the neoplastic cells [11].

A major difficulty is that there is no standardized definition among pathologists; Dunn et al. [12] define PNI as the presence of malignant cells in the perineural space with total or nearly total circumferential nerve involvement on tangential histopathological sections [12]; however both this definition and Liebig's [5] fail to make a clear distinction between perineural dissemination or finding of tumor cells in and around the perineural space without invasion of the nerve fascicle or intraneural dissemination, which may affect the assessment of prognostic influence.

The centrifugal or centripetal spread [13] of the tumor along the nerve is probably the main mechanism related to the increased local recurrence observed in IPN. Although neural spread greater than 2 cm is unusual, it has been described in distances up to 12 cm [13].

## Methods

All studies that evaluated IPN in patients with laryngeal SCC were included. There was no restriction on study design, year of publication, or language. Only studies published with full text were included. Overall survival, disease-free survival and locoregional recurrence were evaluated.

**Fig. 1:**
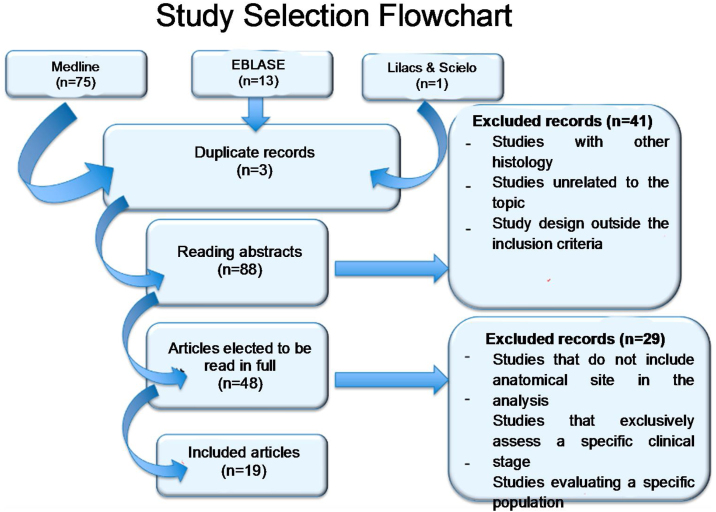
Flow chart.

Excluded were studies where there is no analytical distinction between multiple primary disease sites, non-surgical treatment, studies that primarily evaluate surgical technique, studies that evaluate specific head and neck cancer population, duplicate studies, or studies with replicated casuistry, “in vitro” studies, and studies that primarily evaluate toxicity or quality of life (Fig. 1).

### Statistical analysis

Outcomes were treated as categorical and analyzed with relative risk including 95% Confidence Interval (95% CI). Significant heterogeneity was defined as I^2^ > 50%. A random effect model was used except when statistical heterogeneity was not significant. The funnel plot was used for heterogeneity assessment. Analyses were developed in RevMan 5.4 and R software, in the “Meta-Analysis” package.

## Results

### Two-year overall survival

**Fig. 2:**
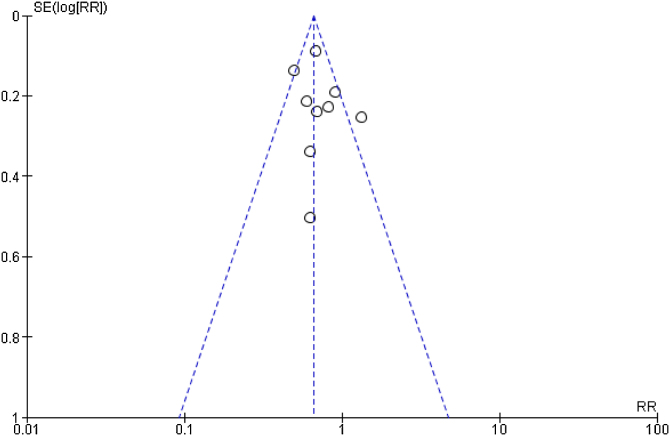
Funnel plot of the 2-year survival meta-analysis.

The funnel plot, used to assess publication bias, identified potential bias (Fig. 2).

**Fig. 3:**
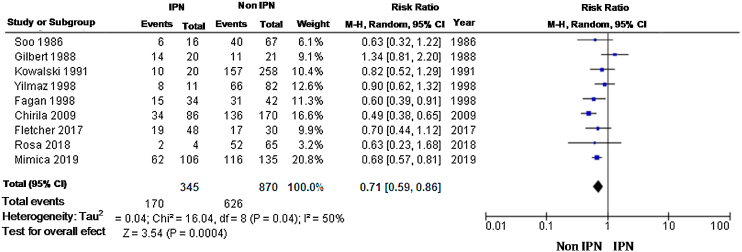
Two-year survival meta-analysis using a random-effect model.

Survival was higher in the group without IPN. A total of 9 studies with 1215 patients were used; the relative risk was 0.66 (95% CI 0.59‒0.74), with a high rate of heterogeneity to fixed model evaluation; therefore, a random model was used, with RR 0.71 (95% CI 0.59‒0.86) (Fig. 3).

### Two-year mortality

**Fig. 4:**
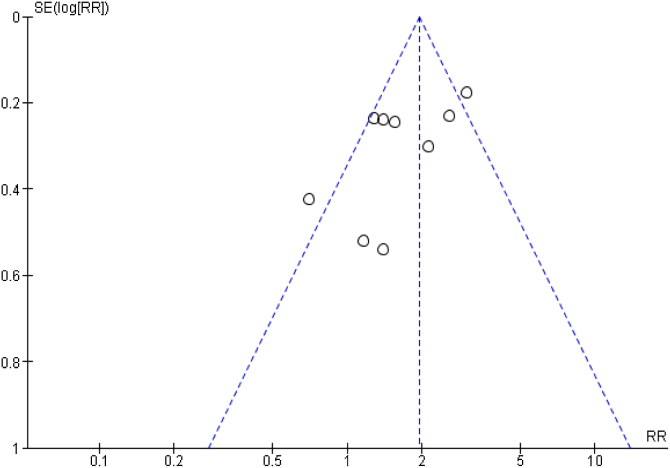
Funnel plot of the 2-year mortality meta-analysis.

The funnel plot, used to assess publication bias, identified potential bias (Fig. 4).

**Fig. 5:**
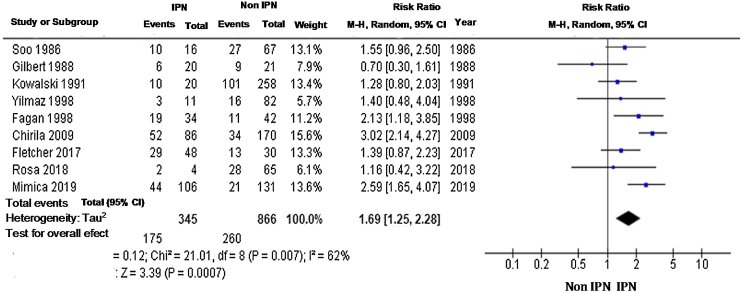
Meta-analysis of 2-year mortality using a random-effect model.

The risk of death is higher in the group with IPN. A total of 9 studies with 1211 patients were used in this evaluation; the relative risk was 1.95 (95% CI 1.64–2.32), with a high rate of heterogeneity to the fixed model evaluation; therefore, it was a random model, with RR 1.69 (95% CI 1.25–2.28) (Fig. 5).

### Two-year disease-free survival

**Fig. 6:**
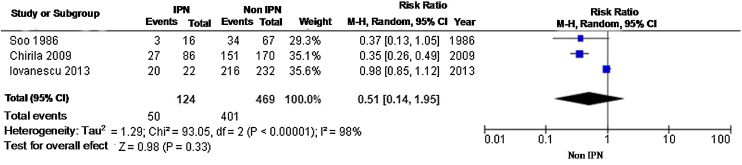
Two-year meta-analysis of DFS using a random-effect model.

A total of 593 patients were evaluated; the relative risk was 0.51 (95% CI 0.42‒0.62), with a high rate of heterogeneity at fixed model evaluation. At random model evaluation, the RR was 0.51 (95% CI 0.14–1.95) (Fig. 6).

### Locoregional relapse

**Fig. 7:**
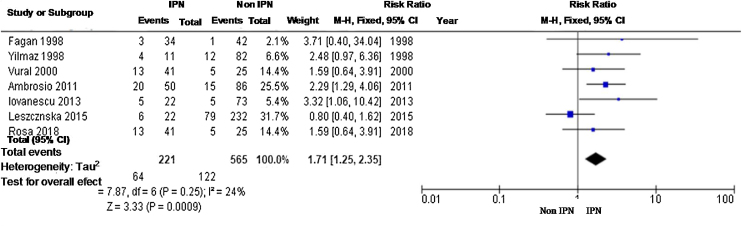
Meta-analysis of locoregional recurrence using a fixed-effect model.

A total of 817 patients were evaluated; the relative risk was 1.71 (95% CI 1.25–2.35), with a low rate of heterogeneity to fixed model evaluation (Fig. 7).

## Discussion

Perineural Invasion (PNI) is predominantly characterized by neoplastic invasion of nerves; it can occur in the absence of lymphatic or vascular invasion. Its propagation does not occur through lymphatic dissemination but rather through molecular mediators that guide these cells through neural invasion.

PNI was first described in head and neck cancer by Cruveilhier [7] in 1835, however, its mechanism of invasion and dissemination remained controversial for more than a century. Only in 1985, Batsakis [8], in his study “Nerves and neurotropic carcinomas”, proposed its definition as “tumoral invasion into, around, and through the nerve”. Since then, this has been the definition used when reporting PNI.

In 2009, Liebig et al., after reviewing the current literature, presented a new definition for PNI as “invasion of one of the three layers of the nerve or involvement of at least one-third of the nerve's circumference” [5]. Since then, this has been the most used definition by most PNI researchers, although some studies still use Batsakis's definition in their analyses.

To address the need for a more uniform definition, we propose a standardized approach that includes histological confirmation, quantitative criteria, and clinical and imaging correlation. This approach aims to reduce variability in study results and improve clinical applicability by providing a clear and detailed definition of PNI.

The presence of PNI significantly alters the therapeutic landscape for patients with head and neck cancers. Recognizing PNI as a high-risk feature necessitates a more aggressive treatment approach, including wider surgical margins, consideration for adjuvant radiation therapy, and the potential for targeted therapies and immunotherapy [14]. Investigating the molecular pathways associated with perineural invasion could unveil potential targets for therapy, enhancing treatment efficacy against PNI-positive tumors.

The detection of PNI, especially involving small-caliber nerves, poses a significant challenge. To enhance the diagnostic accuracy, we propose utilizing a combination of high-resolution MRI with Diffusion-Weighted Imaging (DWI) and Positron Emission Tomography (PET), along with the development of advanced imaging algorithms. These advancements could aid in the early and accurate detection of PNI, improving patient outcomes.

Neoplastic cells tend to concentrate in the perineurium, which is hypovascularized, and can extend up to 12 cm beyond the surgical margin, with skip lesions; therefore, the surgical resection margin is generally insufficient to treat patients who present with previously undetected PNI.

Tumoral growth via neural can occur in two ways:

- Perineural invasion, usually in the smaller nerves, instead of the named nerves. The invasion of the smaller nerves is associated with an increased risk of local recurrence and cervical metastases and is independent of the risk of capsular rupture, a predictor of survival [15]. It is identified microscopically;
- Perineural dissemination, where there is gross invasion of the nerve.

The diagnosis of PNI can be difficult to make, since both pre-operatively and post-operatively there are factors that contribute to this: only 30%–40% of patients presenting with PNI are effectively symptomatic; as well as only 63% of PNI cases can be identified by MRI (MRI identifies massive neural lesions, with indirect signs of invasion, such as enlargement of foramina or increased neural thickness, for example). Such patients are those who already present with gross neural invasion, in tumors of already advanced stage, where PNI will not be the defining factor of prognosis but rather the size of the lesion, in most cases. Moreover, biopsies are usually small in size and with material distortion, which leads to diagnostic difficulty of PNI by the pathologist, frustrating clinical manifestations, only identified in the presence of massive dissemination.

Despite biomarkers such as N-CAM being highly associated with the presence of PNI, they are still not used in routine pre-operative detection. Even after surgical resection, PNI is not frequently identified in the histopathological analysis, which identifies the larger caliber nerves, not paying attention to the smaller caliber nerves, especially if the nerve is not circumferentially involved by the tumor [16].

Regarding diagnostic difficulties in the post-operative period, skip lesions are identified, which can occur frequently, with centimeters of distance, and the presence of perineural atypias, which complicates the identification of neoplasia by the pathologist.

However, even small caliber nerves (<1 mm) are associated with worse locoregional control. Thus, when PNI is identified post-operatively, radiotherapy is indicated, since there is no assurance of surgical margin in PNI [17].

The presence of PNI significantly impacts therapeutic decisions in laryngeal cancer. Given its association with poor prognosis, high loco-regional recurrence, and decreased survival, patients with PNI-positive laryngeal cancer often require more aggressive treatment strategies. This typically involves combining surgery with adjuvant radiotherapy to improve local control and reduce recurrence rates. Studies have shown that PNI is a significant predictor of poor survival outcomes, necessitating a closer clinical and instrumental follow-up in these patients [18], [19].

Advances in imaging techniques have improved the diagnosis of PNI in laryngeal cancer. Narrow Band Imaging (NBI) combined with White Light Endoscopy (WLE) has shown higher sensitivity and specificity in detecting early laryngeal carcinoma compared to WLE alone. NBI enhances the visualization of tumor-specific neoangiogenesis, allowing for better detection of malignant lesions and more accurate assessment of tumor extension [20]. This improved diagnostic accuracy is crucial for early intervention and appropriate treatment planning, potentially leading to better patient outcomes [21], [22].

The meta-analysis identified clinical heterogeneity, leading to statistical heterogeneity, in the prevalence of IPN (ranging from 5.79% to 62.12% across studies), but clearly identifies IPN as an independent negative prognostic factor in laryngeal cancer, both for locoregional recurrence and reduced survival.

The presence of a broad definition of IPN, as well as the non-standardization of its investigation, is responsible for the heterogeneity that has been reported, leading to discrepancy in its incidence in studies, which is not balanced by an increase in casuistry.

Immunohistochemical studies are being carried out to identify IPN, with the search for markers that are associated with its presence; the expression of N-CAM, GDFN, BDNF, NGF shows a relationship with the presence of IPN [14], but they are not yet investigated in routine clinical practice. Interactions between neoplastic cells and the nerve microenvironment are shown to lead to increased tumor aggressiveness; molecules secreted by the tumor and nerve cells cause tumor behavior to change, both in its proliferation and apoptosis and interaction with the nerve.

Patients with IPN may or may not have neurological symptoms, since histologically, the tumor may invade the nerve and interfere with its blood supply, causing local edema, demyelination, and segmental infarction [23]. Furthermore, diagnosis by imaging (MRI) is only made when IPN occurs in large caliber nerves, with bone erosion, widening of the foramen of the skull base, loss of fat in the pterygopalatine fossa, nerve edema can be identified.

Neural involvement usually starts with branches smaller than 1 mm, progressing to larger ones. When IPN is present, surgical margins are no longer controlled by surgery since tumor progression can occur for up to 12 cm beyond its point of origin. The tumor cells tend to concentrate in the perineurium, a poorly vascularized and relatively hypoxic environment, which leads to a relative radio resistance, thus corroborating to the worse prognosis [15] of IPN.

Furthermore, IPN is associated with increased risk of lymph node metastasis (Ambrosio et al. [24] identifies that the presence of IPN is related to corticortin expression as well as the presence of lymph node metastasis; it is inferred that its overexpression promotes cell migration), with the presence of both being related to reduced survival.[6] This is consistent with the poor prognosis of IPN being more prevalent in advanced stage tumors, which in itself is a negative prognostic factor [25].

The outcome of the treatment of laryngeal SCC depends not only on patient selection and quality of treatment provided to the patient, but also on factors related to the tumor and the patient. Demographic, clinical, pathological, and therapeutic factors influence treatment success [3].

Historically, the survival of patients with larynx SCC has been showing a 4% decrease since 1970, while other head and neck neoplasms have shown an increase in survival. Thus, it is important to identify its risk factors in order to intensify treatment in a systematic way.

## Conclusion

IPN is an independent negative prognostic factor in laryngeal cancer, both regarding survival, disease-free survival and locoregional recurrence.

The identification of clinical heterogeneity opens perspectives for standardization of its investigation in further studies, enhancing the understanding and management of PNI in head and neck cancers. A uniform definition of PNI would standardize its diagnosis across studies, improving the comparability of research findings and their clinical applicability. A deeper understanding of PNI's impact on treatment decisions could lead to more personalized and effective therapeutic strategies. Lastly, advancements in imaging techniques are crucial for the early and accurate detection of PNI, particularly in challenging cases involving small-caliber nerves, thereby improving patient outcomes.

## Funding

This research did not receive any specific grant from funding agencies in the public, commercial, or not-for-profit sectors.

## Conflicts of interest

The authors declare no conflicts of interest.
